# Links between the unfolded protein response and the DNA damage response in hypoxia: a systematic review

**DOI:** 10.1042/BST20200861

**Published:** 2021-05-18

**Authors:** Hannah Bolland, Tiffany S. Ma, Syafiq Ramlee, Kristijan Ramadan, Ester M. Hammond

**Affiliations:** MRC Oxford Institute for Radiation Oncology, Department of Oncology, University of Oxford, Old Road Campus Research Building, Oxford OX3 7DQ, U.K.

**Keywords:** DDR, ER stress, hypoxia, radiation, replication stress, UPR

## Abstract

Hypoxia is a feature of most solid tumours and predicts for poor prognosis. In radiobiological hypoxia (<0.1% O_2_) cells become up to three times more resistant to radiation. The biological response to radiobiological hypoxia is one of few physiologically relevant stresses that activates both the unfolded protein and DNA damage responses (UPR and DDR). Links between these pathways have been identified in studies carried out in normoxia. Based in part on these previous studies and recent work from our laboratory, we hypothesised that the biological response to hypoxia likely includes overlap between the DDR and UPR. While inhibition of the DDR is a recognised strategy for improving radiation response, the possibility of achieving this through targeting the UPR has not been realised. We carried out a systematic review to identify links between the DDR and UPR, in human cell lines exposed to <2% O_2_. Following PRISMA guidance, literature from January 2010 to October 2020 were retrieved via Ovid MEDLINE and evaluated. A total of 202 studies were included. LAMP3, ULK1, TRIB3, CHOP, NOXA, NORAD, SIAH1/2, DYRK2, HIPK2, CREB, NUPR1, JMJD2B, NRF2, GSK-3B, GADD45a, GADD45b, STAU1, C-SRC, HK2, CAV1, CypB, CLU, IGFBP-3 and SP1 were highlighted as potential links between the hypoxic DDR and UPR. Overall, we identified very few studies which demonstrate a molecular link between the DDR and UPR in hypoxia, however, it is clear that many of the molecules highlighted warrant further investigation under radiobiological hypoxia as these may include novel therapeutic targets to improve radiotherapy response.

## Introduction

Oxygen homeostasis in eukaryotes is essential in order to maintain aerobic metabolism and intracellular bioenergetics. Due to the high proliferative and metabolic rates observed in malignant cells, tumours rapidly outgrow their oxygen supply leading to regions of hypoxia (insufficient oxygen) [1]. Typically, the following terms are used to define cellular oxygen concentration: normoxia (21% O_2_), tissue normoxia or physoxia (4–7.5% O_2_), hypoxia (1–2% O_2_) and radiobiological hypoxia (<0.1% O_2_) [2–4]. Both hypoxia and radiobiological hypoxia are associated with the stabilisation of the hypoxia-inducible factors (HIF-1/2) [5]. The hypoxic tumour microenvironment drives genomic instability, down-regulates DNA repair and is associated with therapy resistance. Importantly, hypoxic tumours are associated with more aggressive disease, metastasis and poor patient prognosis [6,7].

**Figure 1.**
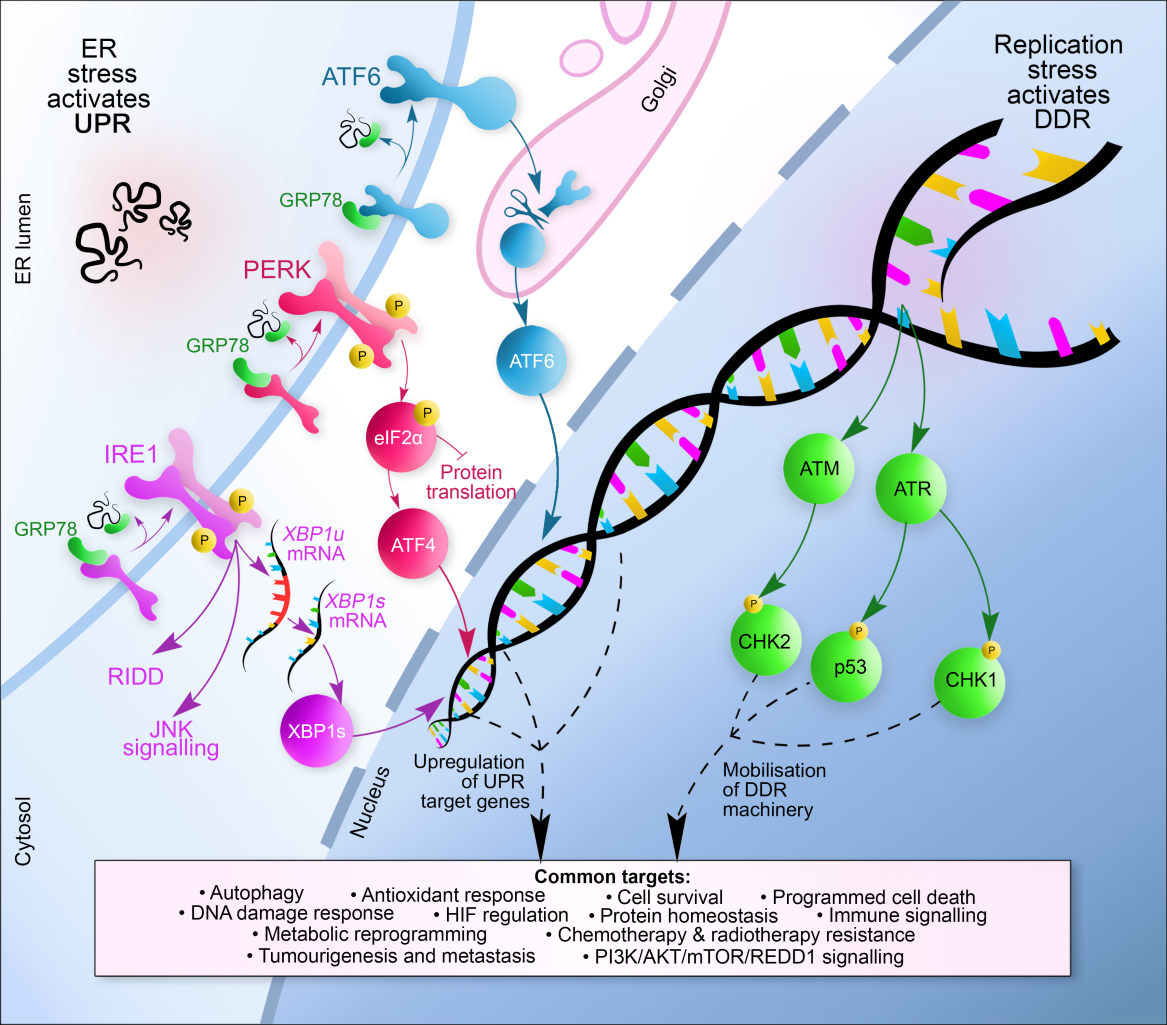
Overview of the hypoxia activated UPR and DDR pathways. The UPR sensors, PERK, IRE1 and ATF4 are held in an inactive state via the binding of GRP78, a chaperone protein and the master regulator of the UPR. Hypoxia-induced ER stress leads to the dissociation of GRP78 from these sensors as GRP78 detects and binds to unfolded proteins. Following activation, PERK phosphorylates eIF2α to inhibit protein synthesis and allows for the transcription of ATF4 which leads to the transcriptional induction of genes associated with apoptosis, autophagy and amino acid metabolism. Activation of IRE1 followed by autophosphorylation activates its RNase activity leading to the alternative splicing of XBP1u to form XBP1s, a transcription factor that controls the transcription of genes encoding proteins involved in protein folding. ATF6 is transported to the Golgi upon activation where it is processed by S1P and S2P generating a cytosolic fragment (ATF6f) which up-regulates the transcription of genes involved in ERAD. Simultaneously, in the cells in S-phase, hypoxia leads to replication stress, manifested by stalled replication forks, decreased origin firing and an accumulation of single-stranded DNA. Hypoxia-induced replication stress activates the DDR pathway which includes both the ATM and ATR kinases. ATM and ATR phosphorylate their target transducers including, but not limited to, Chk1, Chk2 and p53. The transducers then up-regulate effectors, including genes that play a role in cell cycle arrest, apoptosis and DNA repair. The biological endpoints of UPR and DDR signalling are shared and focus on resolving the stress, allowing the cell time to repair and recover or, in the case of extreme stress and irreparable damage inducing cell death.

The biological response to radiobiological hypoxia includes the DNA damage response (DDR) (Figure 1). Deregulation of the DDR is frequent and occurs early in cancer and drives genomic instability [8–10]. Radiobiological hypoxia leads to a replication stress-induced DDR, which is demonstrated by an increase in stalled replication forks, decreased origin firing and significant reduction in DNA replication rates [11,12]. The hypoxia-induced DDR includes the activation of the ATM and ATR kinases, and phosphorylation of their downstream targets (Chk2, KAP1, Chk1, RPA, p53 and H2AX) [13]. Importantly, this DDR occurs in the apparent absence of detectable DNA damage (SSBs or DSBs) [13]. Hypoxia mediated changes to the chromatin also play a critical role in the induction of the DDR, for example ATM activation in hypoxia is linked to both replication stress and trimethylation of histone 3 at lysine 9 (H3K9me3) [14,15].

The unfolded protein response (UPR) is a cyto-protective, adaptive mechanism that maintains protein homeostasis (Figure 1). The UPR is activated upon the detection of misfolded proteins in the endoplasmic reticulum (ER), the main site of folding and maturation for transmembrane, secretory and ER-resident proteins in the cell [16]. Stressors such as hypoxia, viral infection, starvation, calcium depletion, hyper- or hypothermia and acidosis are all known to activate the UPR [17]. Experimentally, thapsigargin and tunicamycin, which cause ER stress by blocking the SERCA calcium pump on the ER membrane or by blocking N-linked glycosylation, respectively are commonly used to pharmacologically induce a UPR [18,19]. Once activated, the UPR inhibits protein synthesis, increases the folding capacity of chaperones, and transcriptionally activates UPR genes that lead to a myriad of outcomes (cell death through apoptosis, pro-survival pathways, autophagy, degradation of permanently misfolded proteins through endoplasmic reticulum-associated degradation (ERAD), amino acid and lipid biosynthesis, and redox homeostasis) [20]. Disulfide bonds occur in a significant proportion of membrane and secreted proteins, through an oxygen-dependent process including ER oxidoreductin (Ero1), and the soluble thiol-disulfide oxireductase protein disulfide isomerase (PDI) [21–23]. Therefore, radiobiological hypoxia leads to an accumulation of misfolded proteins within the ER lumen as a result of an inability to form disulfide bonds [24,25].

Notably, both the DDR and UPR are induced in response to hypoxia; however, most importantly this occurs with the same oxygen dependency. Radiobiological hypoxia (<0.1% O_2_) is required to activate both the DDR and UPR. This suggests that the most therapy resistant fraction of solid tumours has active DNA damage and unfolded protein responses and raises the possibility that the two pathways may interact to lead to biological outcomes that promote tumour survival.

In support of this hypothesis, we recently demonstrated that hypoxia induces expression of the RNA/DNA helicase, senataxin (SETX), through the PERK/ATF4 arm of the UPR. Hypoxia-induced SETX reduces replication stress potentially through the resolution of DNA/RNA hybrids (R-loops) and prevents the accumulation of DNA damage [26]. SETX was also identified through RNA-sequencing as part of an XBP1-dependent gene signature [27]. To our knowledge, this is the most explicit link between the DDR and UPR that has been described in hypoxic conditions.

Direct links between the UPR and DDR under normoxic conditions have been described (recently reviewed in [28]). Indeed, the UPR may play a role in protecting the genome from DNA damage in normoxia. For example, IRE1 has an evolutionarily conserved role in protecting cells exposed to genotoxic stress through controlling the mRNA stability of DDR genes which impacts DNA repair, cell cycle arrest and apoptosis [29]. PERK deficiency leads to an accumulation of ROS and oxidative DNA damage in tumour cells and a Chk2-dependent cell cycle arrest [30]. Thapsigargin-induced UPR leads to the phosphorylation of Chk1 via Claspin1 and slowing of replication forks [31].

Importantly, like hypoxia, exposure to radiation has been shown to lead to both a DDR and UPR [32,33]. While a number of DDR inhibitors have been shown to increase radiosensitivity in both normoxic and hypoxic conditions, the links between the UPR and radiation response are less clear [34]. Studies have shown that inhibiting the UPR can increase radiosensitivity. Specifically, inhibition of PERK or knockdown of ATF4 reduces survival of glioblastoma cells exposed to ionising radiation (IR) [35]. The UPR can activate pro-survival autophagy through the PERK and IRE1 arms following irradiation [33,36]. Exposure to ionising radiation (1 Gy) has been shown to up-regulate UPR gene expression including GRP78, ATF4, CHOP and XP1 in blood samples collected from cancer patients [37]. However, studies also exist which demonstrate that activating the UPR can sensitise cells to ionising radiation. For example, the UPR can suppress DSB repair and sensitise tumour cells to ionising radiation through the proteasomal degradation of Rad51, an essential factor for homologous recombination [38]. Activation of the UPR using the HIV protease inhibitor, nelfinavir, was shown to radiosensitise head and neck cancer cells [39]. UPR activation through the inhibition of the protein disulfide isomerase, PDIA1, decreases DNA repair capacity after irradiation through the down-regulation of DNA repair genes and leads to increased radiosensitivity [40]. This evidence linking the UPR to radiosensitivity highlights an additional example of where the DDR and UPR co-exist.

The DDR and UPR direct a coordinated response to cellular stress that is particularly relevant in hypoxic conditions which induce both replication and ER stress leading to the DDR and UPR, respectively. Moreover, these hypoxic conditions provide the ideal context to uncover UPR-DDR links as it is one of the few physiologically relevant stresses that induces both pathways. This systematic review presents an analysis of published literature in the last decade, for potential links between the DDR and UPR specifically in hypoxic conditions.

## Methods

**Figure 2.**
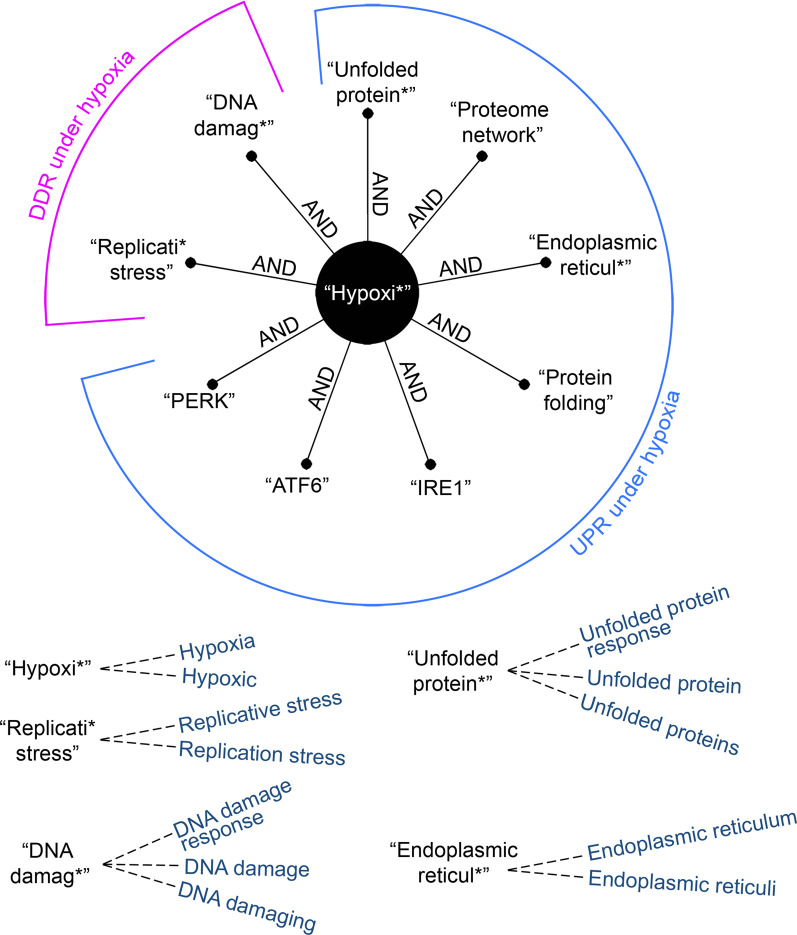
Boolean search strategy used. Relevant and closely related terms were determined and appropriately truncated to account for derivational affixes.

**Figure 3.**
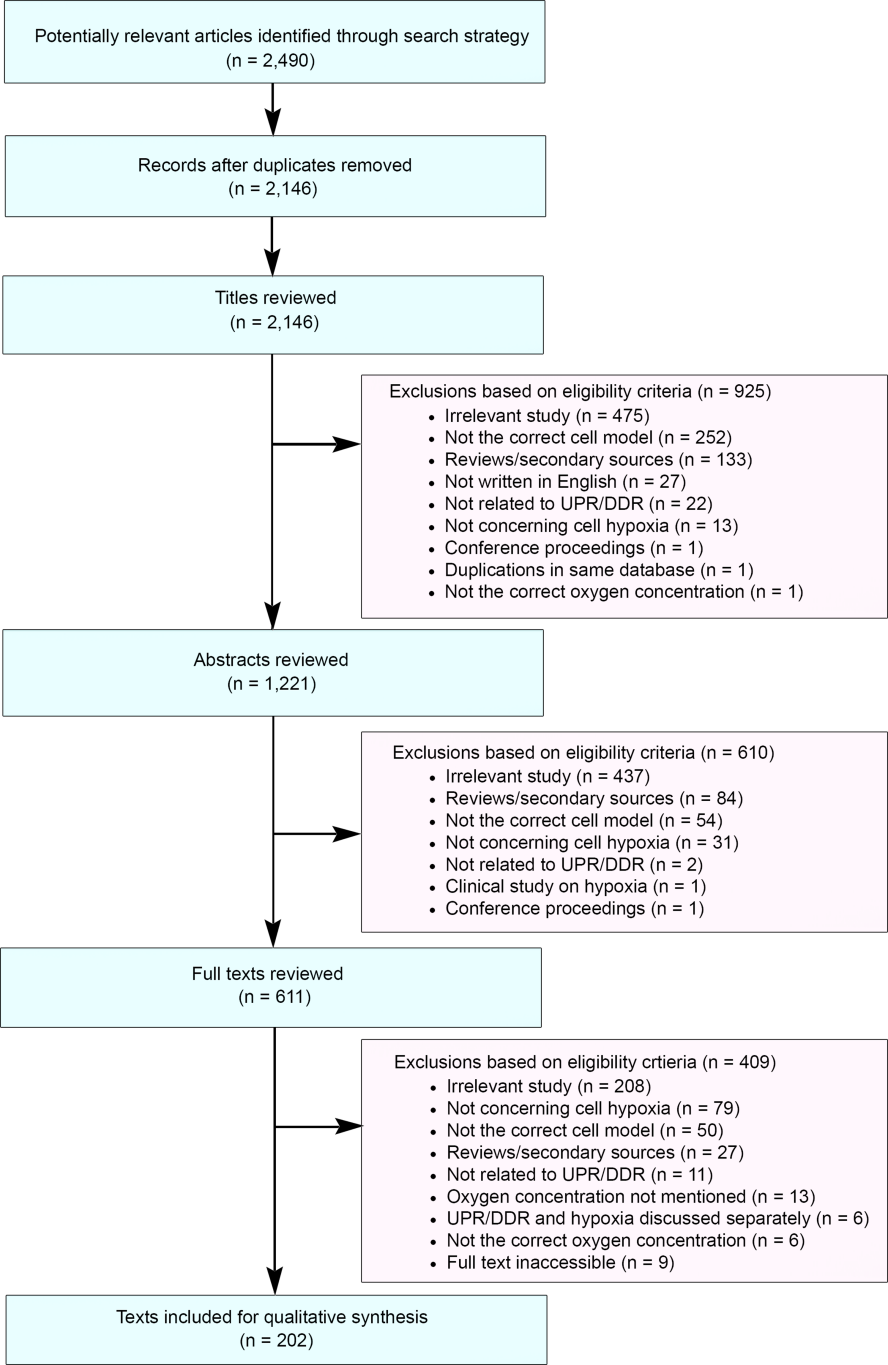
PRISMA flow diagram of the systematic literature exclusion process. Ovid MEDLINE database was used to identify potentially relevant studies published between January 1, 2010 and October 9, 2020.

This systematic review was conducted following the standards set by the PRISMA (Preferred Reporting Items for Systematic Reviews and Meta-Analyses) statement. Literature containing experimental evidence were retrieved via Ovid MEDLINE, following an electronic search restricted to publications between January 1, 2010 and October 9, 2020. This period was chosen to ensure the most recent publications were reviewed but to also limit the number of publications to allow thorough review. To increase the sensitivity of our search, relevant and closely related terms were determined and appropriately truncated to account for derivational affixes (Figure 2). Shortlisted records were initially reviewed for duplicates; remaining materials were then evaluated for suitability at the title, abstract and full-text levels. Study selection was based on eligibility criteria designed around the SPIDER (Sample, Phenomenon of Interest, Design, Evaluation and Research type) framework (Supplementary Table S1). Any paper with hypoxic conditions of <2% O_2_ was included in the screen. We chose to include this broad range of hypoxic conditions because the reported O_2_ level rarely reflects the actual O_2_ concentration in the cell culture dish and in some cases (e.g. after prolonged exposure), the O_2_ concentration may be significantly lower than reported and therefore lead to a DDR/UPR. Large variations in methodology for hypoxia induction were found and include differences in duration, glucose levels in the medium, and surface area of the dish. To reduce subjectivity, the screening was conducted independently by two reviewers (Bolland, H. and Ma, T.), checked by a third reviewer (Ramlee, S.), and any discrepancies between the results of the screens were resolved. The search yielded 2,490 articles for review and, following exclusion, 202 articles remained for further analysis (Figure 3 and Supplementary Table S2).

Data extraction was then carried out to assess study methodology (oxygen concentration and cell lines used), reported observations and identified biological regulators. Papers which were identified as being part of the hypoxic DDR or the hypoxic UPR but not both, were further investigated to determine if by including studies carried out in normoxic conditions links between the UPR and DDR could be determined. One limitation of this methodology is that papers that include experiments carried out in hypoxia but do not contain the key terms in the title or abstract will have not been included in this screen (for example [41]).

## Results

Our systematic approach identified no published studies that have explicitly investigated the links between the DDR and the UPR in hypoxia (<2% O_2_). However, it was clear that the DDR and the UPR interact with many of the same pathways in hypoxia, including: autophagy, HIF regulation, immune signalling, metabolic reprogramming, antioxidant response and PI3K/AKT/mTOR signalling. Through systematic review, we have identified several genes, which although not linked directly to both the hypoxia-induced DDR and UPR, represent credible links between these two pathways. Specifically, we identified genes which have been described to play roles in the hypoxic DDR or UPR but by including some studies in normoxic conditions we found evidence that the genes play roles in both stress response pathways (Supplementary Table S3). The majority of the factors identified are discussed in brief below, organised by the stage/arm of the UPR they are linked to.

## General ER stress induced

Specificity protein 1 (Sp1) is a transcription factor that is overexpressed in several cancers and regulates genes involved in cell cycle and proliferation [42]. Sp1 has been shown to have a dual role in regulating the UPR in Panc-1 cells; it is required for GRP78 activation by ER stress and, in the same cell line, is required for the induction of UPR genes via binding to ER stress response elements (ERSEs) in target gene promoters [43]. Sp1 and HIFs collaborate upon exposure to 1% O_2_ to activate genes needed to adapt to the hypoxic microenvironment [44]. Sp1 knockdown leads to lysosomal membrane permeabilisation, chronic ER stress and cell death due to the inability of cells to activate GRP78 and the UPR [45]. In response to ionising radiation, Sp1 is phosphorylated by ATM and localises with the MRN complex at sites of DNA damage to facilitate DNA repair [46]. Together, these data suggest that inhibition of Sp1 would represent a strategy to radiosensitise hypoxic tumours via inhibition of both the cytoprotective UPR and DDR.

Clusterin (CLU), is a pro-survival, chaperone-like protein, that stabilises unfolded proteins and has been demonstrated to play a role in DNA repair and cell cycle regulation [47,48]. CLU is induced upon genotoxic stress; siRNA inhibition of CLU was found to sensitise cells to a variety of DNA damaging agents including ionising radiation [49–51]. CLU has previously been shown to be induced under 1% O_2_ via direct binding of HIF1α to hypoxic response elements in the CLU promoter [52]. CLU was found to be required for the activation of pro-survival autophagy in human proximal tubular epithelial cells induced by exposure to 1% O_2_. Furthermore, CLU knockdown led to a reduction in UPR related gene expression in hypoxic kidney cells [53]. Given that loss of CLU has been shown to radiosensitise and that it is induced in hypoxia (1% O_2_), it seems likely that inhibition of CLU could be an effective strategy to improve radiation response of hypoxic tumours.

## PERK signalling

Lysosome-associated membrane glycoprotein 3 (LAMP3) expression is controlled by the PERK/ATF4 arm of the UPR. In response to ionising radiation, LAMP3 knockdown was found to reduce the formation of γH2AX foci and the expression of DDR proteins (DNAPK, phosphorylated ATM and ATR) leading to increased radiosensitivity [54]. The LAMP3-dependent impact on the DDR was proposed to be through an autophagy-dependent mechanism. Autophagy has previously been implicated in cellular radiosensitivity through multiple mechanisms including ATM/AMPK/TSC2 signalling (recently reviewed [55]). Our analysis highlighted that LAMP3 was also induced following treatment with tunicamycin and, in a cell line-dependent manner, in hypoxia (0.1% O_2_) [56]. Knockdown of PERK, ATF4 and LAMP3 led to a reduction in breast cancer cell migration, with more pronounced effects observed at 0.1% O_2_ compared with 1% O_2_. LAMP3 knockdown also reduced spheroid invasion in a breast cancer cell line [57]. It seems likely that LAMP3 knockdown may impact the hypoxic DDR and UPR and affect radiosensitivity.

Unc-51 like autophagy activating kinase 1 (ULK1) is a serine-threonine kinase that regulates autophagic flux [58]. In response to DNA damage, ULK1, is a transcriptional target of p53 and enhances the activity of the chromatin-associated enzyme PARP1 leading to sustained autophagy and cell death [59,60]. ULK1 is rapidly induced following exposure to hypoxia (<0.02% O_2_) and is under the control of the PERK arm of the UPR [61]. ULK1 knockdown significantly reduced clonogenic survival of human colorectal and breast cancer cell lines in hypoxia (<0.02% O_2_) but not in normoxic conditions [61]. Given the evidence that ULK1 plays a role in the UPR in hypoxia and is linked to the DDR in normoxia, it seems likely that perturbing ULK1 in hypoxia would impact both the UPR and DDR.

TRIB3 is a pseudo kinase protein, which lacks kinase activity and is overexpressed in several human cancers [62,63]. TRIB3 has been shown to protect HeLa cells from DSBs induced as the result of APOBEC3A-mediated deamination and is part of the CtIP-BRCA1-ATM network regulating the cell cycle, DNA repair and genome stability [64]. TRIB3 has been shown to be induced by both thapsigargin and hypoxia (0.1–0.5% O_2_) in breast cancer cells and was found to co-localise with Pimonidazole (a marker of hypoxia) in breast cancer tissue. In hypoxic conditions, TRIB3 induction was found to be dependent on the PERK/ATF4/CHOP pathway and depletion of TRIB3 reduced cell survival under hypoxic conditions [65]. Interestingly, mass spectrometry has revealed that TRIB3 binds to DNA damage related proteins HMG1, TOP1, Ku80 and multiple members of the DDX family of RNA/DNA helicases [66]. This raises the question of whether inhibition of TRIB3, a UPR induced protein, leads to changes in genomic stability via its interactions with DDR proteins in hypoxia and therefore links the UPR to the protection of the genome in hypoxia.

DNA damage-inducible transcript 3 (DDIT3 or CHOP) was originally identified as a transcription factor responding to UV radiation and has since been shown to be a key effector of the UPR [67,68]. CHOP activity leads to cell cycle arrest and apoptosis following chronic UPR activation through transactivation of targets including BIM, NOXA PUMA, BAD and GADD34 [69,70]. CHOP depletion through siRNA knockdown reduces cell viability in hypoxic (1% O_2_) prostate stromal cells via the inhibition of hypoxia-induced autophagy [71]. NOXA, a CHOP target gene, is responsible for apoptosis and hypoxia-induced cell death, and responds to DNA damage-induced p53 activation [72]. The lncRNA NORAD (non-coding RNA activated by DNA damage) is needed for the assembly of a topoisomerase complex for maintaining genome stability [73]. NORAD has been demonstrated to be hypoxia-inducible (1% O_2_) and is required for full activation of the UPR including CHOP expression [74]. Disruption of CHOP may lead to a reduction in cell death but promote genomic instability; this warrants further investigation under radiobiological hypoxia to determine if this enhances radiosensitivity or otherwise.

SIAH proteins are E3 ubiquitin ligases that modulate the hypoxic response via their degradation of the prolyl hydroxylase, PHD3, leading to HIF1α stabilisation [75]. In addition, SIAH1/2 have been shown to play a role in the DDR via its interactions with p53, HIPK2, ATM and ATR [76]. SIAH1/2 are stress responsive; after DNA damage, SIAH1 expression is induced by p53 and the protein is phosphorylated by ATM/ATR [77]. SIAH1/2 are also part of the UPR, the transcription of SIAH1/2 is dependent on PERK/ATF4 and IRE1α/XBP1 in response to tunicamycin [78]. The induction of SIAH proteins by both the UPR and DDR suggests possible links in hypoxic conditions. For example, in hypoxia (1% O_2_) the dual-specificity protein kinase, DYRK2, is a target of SIAH2-dependent degradation. The consequence of SIAH2-dependent degradation of DYRK2, includes reduced phosphorylation of p53 at residue serine 45 in response to doxorubicin-induced DNA damage in hypoxia [79]. An interesting hypothesis is that the hypoxia (<0.1% O_2_) induced UPR may modulate the balance between SIAH2 and DYRK2 altering p53 dependent apoptosis in hypoxia. SIAH2 was also found to regulate the DDR through Chk2 turnover; inhibition of SIAH2 under 1% O_2_ led to an increase in Chk2 expression [80].

The transcription factor, CREB is overexpressed in many human cancers and regulates the control of genes that function in cell cycle control, DNA repair and metabolism [81]. CREB has previously been demonstrated to regulate the expression of key DDR genes including ATM, ATR and RAD51; for example overexpression of CREB led to an increase in DDR signalling following etoposide treatment [82]. This systematic review highlights that phosphorylation and activation of CREB occurred in response to treatment with either thapsigargin or tunicamycin [83]. The same report described an induction of CREB in response to prolonged treatment (48 hours) at 1% O_2_, although the authors noted the induction of the UPR in response to 1% O_2_ was only moderate in comparison with Thapsigargin or Tunicamycin treatment [83]. These data suggest that CREB activity is induced in response to an UPR and that this would be more evident in radiobiological levels of hypoxia.

NRF2 is the master transcriptional regulator of the antioxidant response [84]. NRF2 has been shown to protect epithelial cells from ionising radiation by enhancing DDR signalling, reducing chromosomal aberrations and releasing DNA replication block [85]. Specifically, after irradiation, NRF2 was found to directly bind to antioxidant response elements (AREs) within the promoter region of the DNA damage sensor, 53BP1 [85]. Results from our analysis found that NRF2 was activated following exposure to hypoxia (24 hours at 1% O_2_) in HepG2 cells. NRF2 was also found to activate ABCB1 mediated chemoresistance via an increase in drug efflux in hypoxia. Indeed, siRNA knockdown of NRF2 increased doxorubicin-induced apoptosis in HepG2 cells exposed to 1% O_2_ [86]. NRF2 has also been shown to be up-regulated in response to anoxia and thapsigargin treatment in retinal pigment epithelial cells and concomitantly with the UPR under severe hypoxia but not mild hypoxia (4% O_2_) in human lens epithelial cells [87,88]. Hypoxia, somewhat paradoxically, leads to an increase in mitochondrial ROS, leading to dissociation of NRF2 from its negative regulator Keap1, allowing it to translocate to the nucleus to regulate the transcription of antioxidant response genes [89,90]. Disruption of NRF2 signalling in radiobiological hypoxia may radiosensitise cells through its interaction with both the DDR and UPR.

The serine-threonine kinase, GSK-3β, contributes to the hypoxia-induced UPR (<1% O_2_) through down-regulation of γ-Taxilin, an inhibitor of ATF4 activity [91]. GSK-3β has been shown to contribute to UPR-induced apoptosis via PP2A/AKT-dependent GSK-3β activation. This interaction occurs on a cytoplasmic ATM platform. Exposure to 1% O_2_ was found to increase the expression of PP2A in ATM precipitates in 293T cells [41]. In addition, GSK-3β has been shown to translocate from the cytoplasm to the nucleus to induce DNA DSB repair by phosphorylating 53BP1 after irradiation and has been described as indispensable for DNA DSB repair [92]. The role of GSK-3β in the radiation response in hypoxic conditions which also lead to repression of the main DNA repair pathways is likely to yield interesting findings.

### IRE1α signalling

SRC is a protein-tyrosine kinase that is overexpressed in a variety of cancers and induced in hypoxia [93,94]. Inhibition of c-SRC radiosensitised glioblastoma cells exposed to 1% O_2_ and this was attributed to comprised DNA repair as indicated by sustained γH2AX staining [95]. SRC is activated by the UPR, forms a complex with IRE1α and acts to relocate ER chaperones to the cell surface [96]. In addition, SRC has also been implemented in regulation of the DDR via termination of the ATR-Chk1 dependent G2 DNA damage checkpoint [97]. Together, these findings suggest that inhibition of c-SRC may have profound impact on the radiosensitivity of cells experiencing radiobiological hypoxia i.e. the most therapy resistant tumour fraction.

Hexokinase 2 (HK2) the rate limiting enzyme in glycolysis is often overexpressed in cancers [98]. HK2 is induced following exposure to 1% O_2_ and treatment with tunicamycin in an XBP1-dependent manner [99]. Loss of HK2 expression led to a reduction in glioma cell viability in hypoxia [99]. Furthermore, loss of HK2 led to increased DNA damage measured via comet assay in response to IR in U87 and GS2 cells [100]. These data suggest that HK2 would likely also be induced under radiobiological hypoxia and its inhibition could radiosensitise through increased DNA damage.

Caveolin-1 (Cav1) is a membrane protein and the main component of caveolae which are cholesterol enriched invaginations of the plasma membrane that are important for endocytosis, cell transport and cell signalling. Cav1 is overexpressed in a variety of cancers [101]. Cav1 expression suppresses UPR activation in melanoma cells following exposure to hypoxia (1% O_2_) via direct binding to and repression of IRE1α [102]. Knockdown of Cav1 increased UPR activation and led to an increase in IR-induced ssDNA and γH2AX alongside a reduction in phospho-ATM and DSB repair by HR [103].

## ATF6 signalling

Cyclophilin B (CypB) is a molecular chaperone that acts to fold and process newly synthesised proteins [104]. CypB is up-regulated in hypoxia by ATF6 (0.1% O_2_) and cooperates with p300 to modulate the ubiquitination and degradation of CHOP under hypoxic conditions [105,106]. Knockdown of CypB led to an increase in hypoxia induced apoptosis at 0.1% O_2_ [106]. CypB knockdown supressed p53 induction and sensitised cells to daunorubicin [104].

## Conclusion

There are clear mechanistic and functional links between the DDR and UPR [28]. Hypoxia is one of few physiologically relevant stresses to lead to both a UPR and DDR and yet, surprisingly, there are only isolated reports of links between these two pathways. Furthermore, in those that have been investigated in hypoxia, there is a lack of consensus regarding the appropriate oxygen concentration to be used for the study of the DDR and UPR. Of note, the majority of the papers identified in this review focus on cancer, however, many reports identified in the initial screen investigated ischemia-reperfusion injury, stroke, obstructive sleep apnoea and hypertension. Due to exclusion criteria used in this review, these papers were not included as they were predominantly carried out in mouse or rat cell lines. It is likely that further links between the hypoxic DDR and UPR could be identified by also considering these pathophysiological conditions. Overall, the genes and proteins that have been identified from this review demonstrate that significant links between the hypoxic UPR and DRR are likely and warrant further investigation in this context. Most importantly, research in this area could identify therapeutic strategies which could be used to target the most therapy-resistant fraction of solid tumours.

## Perspectives

- Both the UPR and DDR pathways have a significant impact on the cellular response to hypoxia.
- Links between the two pathways are emerging using pharmacological induction of either pathway.
- Further research is highly likely to identify mechanistic and functional links between the UPR and DDR which are important in hypoxia.

## Open Access

Open access for this article was enabled by the participation of University of Oxford in an all-inclusive *Read & Publish* pilot with Portland Press and the Biochemical Society under a transformative agreement with JISC.

## Abbreviations

AREs: antioxidant response elements
CLU: Clusterin
ER: endoplasmic reticulum
ERAD: endoplasmic reticulum associated degradation
HIF: hypoxia inducible factors
HK2: hexokinase 2
LAMP3: Lysosome-associated membrane glycoprotein 3
SETX: senataxin
ULK1: autophagy activating kinase 1
UPR: unfolded protein response
