# Supplementary material for: Links between the unfolded protein response and the DNA damage response in hypoxia: a systematic review
Source: Biochem Soc Trans. 2021 May 18;49(3):1251–63. doi: 10.1042/BST20200861 (PMC8286837; doi:10.1042/BST20200861)
Supplement: Supplementary Tables S1-S3 [file BST-49-1251-s1.pdf]

Table S1. **Eligibility criteria used to evaluate papers for inclusion or exclusion in review.** Inclusion and exclusion criteria were determined based on the SPIDER framework.

	**Inclusion criteria**	**Exclusion criteria**
Sample	Human cell model	Non-human cell model
Phenomenon of Interest	Activation of DDR and/or UPR (proteins, genes, transcripts) under hypoxia	Study not relevant to UPR and/or DDR and/or hypoxia
Design	Cell lines exposed to hypoxia at <2% O_2 concentration	Cell lines not exposed to relevant oxygen concentration (>2% O_2); Exact oxygen concentration not reported
Evaluation	Link between UPR and DDR (alluded or explicit)	Inexplicit link between UPR and DDR; Pathways discussed separately in paper
Research type	Empirical research	Secondary sources and non-empirical texts (i.e. review papers, textbook chapters, conference proceedings); Non-English texts

Table S2. **Papers identified from systematic review.**
Following exclusion according to Table S1, 202 articles remained and were reviewed further

Author	Year Published	Title	Journal	Oxygen Concentration (%)	Cell Line(s) Used	Biological Molecule(s) Examined
Cam, H. et al.	2010	mTORC1 signaling under hypoxic conditions is controlled by ATM-dependent phosphorylation of HIF-1alpha	*Mol Cell*	0.2	HEK, ATM-deficient fibroblasts	mTOR, HIF, ATM, REDD1
Hegan, D. et al.	2010	Inhibition of poly(ADP-ribose) polymerase down-regulates BRCA1 and RAD51 in a pathway mediated by E2F4 and p130	*Proc Natl Acad Sci USA*	0.1	A549, RKO	PARP, BRCA1, RAD51, E2F4, p130
Lou, J. et al.	2010	Inhibition of hypoxia-inducible factor-1alpha (HIF-1alpha) protein synthesis by DNA damage inducing agents	*PLoS One*	1	HEK	p53, HIF, eIF2α, CDK
Moehlenbrink, J. et al.	2010	Hypoxia suppresses chemotherapeutic drug-induced p53 Serine 46 phosphorylation by triggering HIPK2 degradation	*Cancer Let*	0.2 2	H1299, HepG2, MCF7, U2OS	p53, HIPK2, Siah1
Pires, I. et al.	2010	Exposure to acute hypoxia induces a transient DNA damage response which includes Chk1 and TLK1	*Cell Cycle*	0.02	RKO	Chk1, TLK1, RAD51
Pires, I. M. et al.	2010	Effects of acute versus chronic hypoxia on DNA damage responses and genomic instability	*Cancer Res*	<0.02	RKO, HCT116, U2OS	E2F, MCF6, RPA, p53
Regazzetti, C. et al.	2010	Insulin induces REDD1 expression through hypoxia-inducible factor 1 activation in adipocytes	*J Biol Chem*	1	Human adipocytes (Biopredic)	REDD1, mTOR, HIF, PI3K
Ghosh, R. et al.	2010	Transcriptional regulation of VEGF-A by the unfolded protein response pathway	*PLoS One*	0.5	HepG2	VEGFA, ATF6, IRE1/XBP1, PERK
Lee, J. H. et al.	2010	Retinoic acid and its binding protein modulate apoptotic signals in hypoxic hepatocellular carcinoma cells	*Cancer Lett*	1	Huh7, SNU-671	CRABP-II
Pereira, E. R. et al.	2010	Transcriptional and post-transcriptional regulation of proangiogenic factors by the unfolded protein response	*PLoS One*	1	DAOY	VEGFA, FGF8, IL8, XBP1, ATF4
Rouschop, K. et al.	2010	The unfolded protein response protects human tumor cells during hypoxia through regulation of the autophagy genes MAP1LC3B and ATG5	*J Clin Invest*	<0.1	HCT116, U373	MAP1LC3B, ATG5, PERK, ATF4, CHOP
Rzymski, T. et al.	2010	Regulation of autophagy by ATF4 in response to severe hypoxia	*J Clin Invest*	<0.1	MCF7	ATF4
Yanagisawa, K. et al.	2010	Novel metastasis-related gene CIM functions in the regulation of multiple cellular stress-response pathways	*Cancer Res*	0.5	NCI-H460, 293T	CIM, HIF1α, OS-9, PHD, GRP78
Papadakis, A. et al.	2010	eIF2{alpha} Kinase PKR modulates the hypoxic response by Stat3-dependent transcriptional suppression of HIF-1{alpha}	*Cancer Res*	*1.0*	H1299	PKR, STAT, HIF
Bouquet, F. et al.	2011	A DNA-dependent stress response involving DNA-PK occurs in hypoxic cells and contributes to cellular adaptation to hypoxia	*J Cell Sci*	0.1 1	HeLa, U87	DNA-PK, XCRR4 DNA ligase,
Harding, S. et al.	2011	ATM-dependent phosphorylation of 53BP1 in response to genomic stress in oxic and hypoxic cells	*Radiother Oncol*	0.01	HCT116	53BP1, p53
Lai, L. et al.	2011	Down-regulation of NDRG1 promotes migration of cancer cells during reoxygenation	*PLoS One*	0.5	MCF7	NDRG1, HIF, c-MYC, n-MYC

Moon, S. et al.	2011	The expression of human papillomavirus type 16 (HPV16 E7) induces cell cycle arrest and apoptosis in radiation and hypoxia resistant glioblastoma cells	*Mol Med Rep*	0	U87MG	HPV16 E7, p53, Rb, E2F1
Ramaekers, C. et al.	2011	Hypoxia disrupts the Fanconi anemia pathway and sensitizes cells to chemotherapy through regulation of UBE2T	*Radiother Oncol*	0.02	HT29, DU145, MCF7, MDA-MD-468, U373, HCT116, HeLa, ME180,SiHa	UBE2T, HIF, FANCD2
Butler, P. et al.	2011	Unfolded protein response regulation in keloid cells	*J Surg Res*	<0.2	Keloid and normal fibroblasts	PERK, IRE1, ATF6
Fatma, N. et al.	2011	Deficiency of Prdx6 in lens epithelial cells induces ER stress response-mediated impaired homeostasis and apoptosis	*J Surg Res*	1	Human lens (Lions Eye Bank)	PRDX6, PERK, IRE1, ATF6
Roberts, H. et al.	2011	Colon tumour cells increase PGE(2) by regulating COX-2 and 15-PGDH to promote survival during the microenvironmental stress of glucose deprivation	*Carcinogenesis*	1	HT29	PGE2, COX2, 15-PGDH, PI3K, AKT
Tamama, K. et al.	2011	Differential roles of hypoxia inducible factor subunits in multipotential stromal cells under hypoxic condition	*J Cell Biochem*	1	Bone marrow multipotential stromal cells (MSCs) (Lonza)	HIF
Wennemers, M. et al.	2011	Tribbles homolog 3 denotes a poor prognosis in breast cancer and is involved in hypoxia response	*Breast Cancer Res*	0.1	MDA-MB-231	TRIB3, PERK, CHOP, ATF4
Brockmeier, U. et al.	2011	The function of hypoxia-inducible factor (HIF) is independent of the endoplasmic reticulum protein OS-9	*PLoS One*	1	U2OS, HEK293, HEK293T, HeLa, MC-7, UT7, Hep3B	OS-9, HIF, PHD2, PHD3
Miyamoto, N. et al.	2011	Transcriptional regulation of activating transcription factor 4 under oxidative stress in retinal pigment epithelial ARPE-19/HPV-16 cells	*Invest Ophthalmol Vis Sci*	0	ARPE-19/HPV-16	ATF4, NRF2
Van den Beucken, T. et al.	2011	Translational control is a major contributor to hypoxia induced gene expression	*Radiother Oncol*	<0.02	DU145	PERK, eIF2α, mTOR, 4E-BP1, EIF4F
Hamidi, T. et al.	2012	Nupr1-aurora kinase A pathway provides protection against metabolic stress-mediated autophagic-associated cell death	*Clin Cancer Res*	0.2	MiaPaca2	Nupr1, Aurka
Kumareswaran, R. et al.	2012	Chronic hypoxia compromises repair of DNA double-strand breaks to drive genetic instability	*J Cell Sci*	0 0.2	GM05757	gH2AX, 53BP1, ATM
Leontieva, O. et al.	2012	Hypoxia suppresses conversion from proliferative arrest to cellular senescence	*Proc Natl Acad Sci U S A*	0.2	MEL10	mTOR, p21, p53, nutlin-3a
Perez, M. et al.	2012	Mutual regulation between SIAH2 and DYRK2 controls hypoxic and genotoxic signaling pathways	*J Mol Cell Biol*	1	U2OS, HeLa, H1299	SIAH2, DYRK2,
Pires, I. M. et al.	2012	Targeting radiation-resistant hypoxic tumour cells through ATR inhibition	*Br J Cancer*	<0.02	RKO, HCT116, DLD1	ATR, H2AX, KAP1
Sermeus, A. et al.	2012	Hypoxia-induced modulation of apoptosis and BCL-2 family proteins in different cancer cell types	*PLoS One*	1	HepG2, A549, MDA-MB231, Hep3B, U2OS, HT-29, PC-3	BCL2, NOXA, BAD, p53
Urano, M. et al.	2012	The effect of DN (dominant-negative) Ku70 and reoxygenation on hypoxia cell-kill: evidence of hypoxia-induced potentially lethal damage	*Int J Radiat Biol*	0 0.1 0.5	HCT8, HT29	Ku70,
Xie, Y. et al.	2012	SirT1 regulates radiosensitivity of hepatoma cells differently under normoxic and hypoxic conditions	*Cancer Sci*	1	HepG2, SK-Hep-1	SIRT1, CMYC, P53

Zhang, T. et al.	2012	Silencing thioredoxin induces liver cancer cell senescence under hypoxia	*Hepatol Res*	1			MHCC97H	TXN, p53
Bouvier, N. et al.	2012	The unfolded protein response regulates an angiogenic response by the kidney epithelium during ischemic stress	*J Biol Chem*	0.1			HK-2	VEGFA, bFGF, ANG, HIF1α, PERK, IRE1, ATF6
Elanchezhian, R. et al.	2012	Low glucose under hypoxic conditions induces unfolded protein response and produces reactive oxygen species in lens epithelial cells	*Cell Death Dis*	1	<0.14		SRA 01/04	Nrf2, PERK
Munksgaard Persson, M. et al.	2012	HIF-2alpha expression is suppressed in SCLC cells, which survive in moderate and severe hypoxia when HIF-1alpha is repressed	*Cell Death Dis*	1	0		U1690, U1906, U2020, U1285, U1568	HIF2α, XBP1
Rzymski, T. et al.	2012	The unfolded protein response controls induction and activation of ADAM17/TACE by severe hypoxia and ER stress	*Oncogene*	0	<0.1		HeLa, HEF, HEK293, U87, MCF7	ADAM17/TACE, TNFα, TNFR, HIF1α, PERK/eIF2α /ATF4, ATF6,
Tang, X. et al.	2012	Functional interaction between responses to lactic acidosis and hypoxia regulates genomic transcriptional outputs	*Cancer Res*	1			MCF7	ATF4
Yu, S. J. et al.	2012	Enhancement of hexokinase II inhibitor-induced apoptosis in hepatocellular carcinoma cells via augmenting ER stress and anti-angiogenesis by protein disulfide isomerase inhibition	*J Bioenerg Biomembr*	1			Huh7, SNU-761	Hypoxic induction of PDI
Ding, G. et al.	2013	HIF1-regulated ATRIP expression is required for hypoxia induced ATR activation	*FEBS Lett*	0.1			HeLa, MCF7, HepG2, 293T	ATRIP
Fallone, F. et al.	2013	ATR controls cellular adaptation to hypoxia through positive regulation of hypoxia-inducible factor 1 (HIF-1) expression	*Oncogene*	0.1			U20S	ATM, DNA-PK, ATR, CHK1
Hasvold, G. et al.	2013	The efficacy of CHK1 inhibitors is not altered by hypoxia, but is enhanced after reoxygenation	*Mol Cancer Ther*	0.02	0.2	1	U20S, HCT116	CHK1, γH2AX
Olcina, M. et al.	2013	Replication stress and chromatin context link ATM activation to a role in DNA replication	*Mol Cell*	0.1			RKO	ATM
Pan, J. et al.	2013	A kinome-wide siRNA screen identifies multiple roles for protein kinases in hypoxic stress adaptation, including roles for IRAK4 and GAK in protection against apoptosis in VHL-/- renal carcinoma cells, despite activation of the NF-kappaB pathway	*J Biomol Screen*	0.3			SW480	IRAK4, GAK, NF-κB
Selvakumaran, M. et al.	2013	Autophagy inhibition sensitizes colon cancer cells to antiangiogenic and cytotoxic therapy	*Clin Cancer Res*	0.1			HT29, HCT116, HCT15, SW620, KM12	Beclin1, LC3, p62, ATG5
Sermeus, A. et al.	2013	Differential effect of hypoxia on etoposide-induced DNA damage response and p53 regulation in different cell types	*J Cell Physiol*	1			HEPG2, A549	p53, CHK2, H2AX
Storci, G. et al.	2013	Slug/beta-catenin-dependent proinflammatory phenotype in hypoxic breast cancer stem cells	*Am J Pathol*	1			MCF7	Slug/β-catenin, TNF, IL8
Tong, Y. et al.	2013	HIF1 regulates WSB-1 expression to promote hypoxia-induced chemoresistance in hepatocellular carcinoma cells	*FEBS Lett*	1			HepG2	WSB-1, HIPK2
Wrann, S. et al.	2013	HIF mediated and DNA damage independent histone H2AX phosphorylation in chronic hypoxia	*Biol Chem*	0.2			HEK	H2AX, HIF1, HIF2

Zhu, Y. et al.	2013	Involvement of decreased hypoxia-inducible factor 1 activity and resultant G1-S cell cycle transition in radioresistance of perinecrotic tumor cells	*Oncogene*	<0.02		HEK, HeLa	HIF, p27
Blaustein, M. et al.	2013	Modulation of the Akt pathway reveals a novel link with PERK/eIF2alpha, which is relevant during hypoxia	*Oncogene*	<0.1		HEK293T	Akt, PERK
Chiang, C. K. et al.	2013	Endoplasmic reticulum stress signal impairs erythropoietin production: a role for ATF4	*Am J Physiol Cell Physiol*	1		HepG2	HIF1a, ATF4, EPO, GRP78
Cojocari, D. et al.	2013	New small molecule inhibitors of UPR activation demonstrate that PERK, but not IRE1alpha signaling is essential for promoting adaptation and survival to hypoxia	*Radiother Oncol*	0.2		KP4, HCT116	PERK, IRE1
Grkovic, S. et al.	2013	IGFBP-3 binds GRP78, stimulates autophagy and promotes the survival of breast cancer cells exposed to adverse microenvironments	*Oncogene*	1		MCF7	IGFBP-3, GRP78
Mellor, P. et al.	2013	CREB3L1 is a metastasis suppressor that represses expression of genes regulating metastasis, invasion, and angiogenesis	*Mol Cell Biol*	1		MCF7, MDA-MB-435	CREB3L1
Mujcic, H. et al.	2013	Hypoxic activation of the PERK/eIF2alpha arm of the unfolded protein response promotes metastasis through induction of LAMP3	*Clin Cancer Res*	0		ME180	LAMP3, PERK/eIF2α
Nagelkerke, A. et al.	2013	Hypoxia stimulates migration of breast cancer cells via the PERK/ATF4/LAMP3-arm of the unfolded protein response	*Breast Cancer Res*	1		MDA-MB-231	PERK/ATF4/LAMP3
Rouschop, K. et al.	2013	PERK/eIF2alpha signaling protects therapy resistant hypoxic cells through induction of glutathione synthesis and protection against ROS	*Proc Natl Acad Sci U S A*	<0.02	<0.2	U373	PERK/eIF2α, HIF1
Schaaf, M. et al.	2013	The autophagy associated gene, ULK1, promotes tolerance to chronic and acute hypoxia	*Radiother Oncol*	<0.02	<0.2	Prrimary human head and neck squamous cell carcinoma (HNSCC) xenografts	ULK1
Zhang, H. et al.	2013	Effects of hypoxia on the proliferation, mineralization and ultrastructure of human periodontal ligament fibroblasts in vitro	*Exp Ther Med*	1	2	Human periodontal ligament fibroblasts (HPLFs) (donors)	Alkaline phosphatase (ALP)
Koritzinsky, M. et al.	2013	Two phases of disulfide bond formation have differing requirements for oxygen	*J Cell Biol*	0		HepG2, HCT116, HeLa	-
Askautrud, H. et al.	2014	Global gene expression analysis reveals a link between NDRG1 and vesicle transport	*PLoS One*	1		ME16C2, SUM102	NDRG1
Chan, N. et al.	2014	Hypoxia provokes base excision repair changes and a repair-deficient, mutator phenotype in colorectal cancer cells	*Mol Cancer Res*	0.2		RKO	MYH, OGG1, MTH1, APE1, PCNA, POLD, FEN1, DNA POLB, and RPA
Chen, L. et al.	2014	Jumonji domain-containing protein 2B silencing induces DNA damage response via STAT3 pathway in colorectal cancer	*Br J Cancer*	1		CRC	STAT3, JMJD2B
Kilic Eren, M. et al.	2014	The role of hypoxia inducible factor-1 alpha in bypassing oncogene-induced senescence	*PLoS One*	1		IMR-90, BJ	RasV12, p53, $p21^{CIP1}$ and $p16^{INK4a}$, HIF

Possik, P. et al.	2014	Parallel in vivo and in vitro melanoma RNAi dropout screens reveal synthetic lethality between hypoxia and DNA damage response inhibition	*Cell Rep*	1.0		Human melanoma xenografts	CHEK1, CHEK2, ATM
Raz, S. et al.	2014	Severe hypoxia induces complete antifolate resistance in carcinoma cells due to cell cycle arrest	*Cell Death Dis*	0.1		DLD1, HCT116	-
Scanlon, S. et al.	2014	Hypoxic stress facilitates acute activation and chronic downregulation of fanconi anemia proteins	*Mol Cancer Res*	0.1	1	HeLa, A549, MCF7	FANCD2, FANCI, γH2AX, BRCA1 and RAD51
Wang, Y. et al.	2014	Gemcitabine induces poly (ADP-ribose) polymerase-1 (PARP-1) degradation through autophagy in pancreatic cancer	*PLoS One*	1		KLM1	PARP, MEK, ERK, LC3
Wu, M. et al.	2014	Dynamic regulation of Rad51 by E2F1 and p53 in prostate cancer cells upon drug-induced DNA damage under hypoxia	*Mol Pharmacol*	0.2		PC3, DU145, LNCaP, Vcap	RAD51, p53, E2F1
Zhang, L. et al.	2014	Inhibition of KAP1 enhances hypoxia-induced Kaposi's sarcoma-associated herpesvirus reactivation through RBP-Jkappa	*J Virol*	1		Primary effusion lymphoma (PEL), HEK293	KAP1, HIF1α, Kaposi's sarcoma-associated herpesvirus (KSHV)
Chen, X. et al.	2014	XBP1 promotes triple-negative breast cancer by controlling the HIF1alpha pathway	*Nature*	0.1		Triple negative breast cancer, MDA-MB-231, Hs578T	XBP1, HIF1α
Lee, D. et al.	2014	Hyperoxia resensitizes chemoresistant glioblastoma cells to temozolomide through unfolded protein response	*Anticancer Res*	1		U87, U251	P4HB
Pereira, E. et al.	2014	Endoplasmic reticulum (ER) stress and hypoxia response pathways interact to potentiate hypoxia-inducible factor 1 (HIF-1) transcriptional activity on targets like vascular endothelial growth factor (VEGF)	*J Biol Chem*	1		NB1691	HIF1, VEGF, ATF4
Pi, L. et al.	2014	Knockdown of glucose-regulated protein 78 abrogates chemoresistance of hypopharyngeal carcinoma cells to cisplatin induced by unfolded protein in response to severe hypoxia	*Oncol Lett*	<0.1	1	FaDu	GRP78
Vlaminck, B. et al.	2014	Effects of copper sulfate-oxidized or myeloperoxidase-modified LDL on lipid loading and programmed cell death in macrophages under hypoxia	*Hypoxia (Auckl)*	1		Macrophages	-
Jeong, K. et al.	2014	Cyclophilin B is involved in p300-mediated degradation of CHOP in tumor cell adaptation to hypoxia	*Cell Death Differ*	0.1		HeLa, HEK293, AGS	CypB, CHOP, p300, ATF6
Adam, M. et al.	2015	SIAH ubiquitin ligases regulate breast cancer cell migration and invasion independent of the oxygen status	*Cell Cycle*	1		MCF7	SIAH, p27
Allen, C. et al.	2015	DNA Damage Response Proteins and Oxygen Modulate Prostaglandin E2 Growth Factor Release in Response to Low and High LET Ionizing Radiation	*Front Oncol*	1.0		HeLa, HT1080, HCT116, MCF7, BJ1hTERT	PGE2, COX1–2
Bigot, N. et al.	2015	ING1b negatively regulates HIF1alpha protein levels in adipose-derived stromal cells by a SUMOylation-dependent mechanism	*Cell Death Dis*	1		Human adiopcyte tissue samples, U2OS	ING1b, PIAS4
Bigot, N. et al.	2015	Hypoxia Differentially Modulates the Genomic Stability of Clinical-Grade ADSCs and BM-MSCs in Long-Term Culture	*Int J Biochem Cell Biol*	1		Human adiopcyte tissue samples	Ku80, TP53BP1, BRCA1, and RAD51
Kuo, Y. et al.	2015	Nijmegen breakage syndrome protein 1 (NBS1) modulates hypoxia inducible factor-1alpha (HIF-1alpha)	*Int J Biochem Cell Biol*	1		MDA-MB-231	MRE11–RAD50–NBS1

		stability and promotes in vitro migration and invasion under ionizing radiation					
Leszczynska, K. et al.	2015	Hypoxia-induced p53 modulates both apoptosis and radiosensitivity via AKT	*J Clin Invest*	<0.1		H1299	p53, AKT, INPP5D, PHLDA3
Liu, Y. et al.	2015	Hypoxia induced HMGB1 and mitochondrial DNA interactions mediate tumor growth in hepatocellular carcinoma through Toll-like receptor 9	*J Hepatol*	1		Hepa1-6, Huh7	HMGB1, TLR9
Shi, S. et al.	2015	Resistance of SMMC-7721 hepatoma cells to etoposide in hypoxia is reversed by VEGF inhibitor	*Mol Med Rep*	0.6		SMMC-7721	VEGF, Bcl-2, procaspase 3, cyclin B1 and Cdc2, NFKB
Thurmond, P. et al.	2015	Structural modifications of the prostate in hypoxia, oxidative stress, and chronic ischemia	*Korean J Urol*	2		Human prostate smooth muscle cells (SMCs), epithelial cells (ECs), and stromal cells (SCs)	Oxidate damage markers
Vasilevskaya, I. et al.	2015	Inhibition of JNK Sensitizes Hypoxic Colon Cancer Cells to DNA-Damaging Agents	*Clin Cancer Res*	0.05		HT29	JNK
Chatterjee, N. et al.	2015	Environmental stress induces trinucleotide repeat mutagenesis in human cells	*Proc Natl Acad Sci U S A*	1.0		HEK293	HIF1, DHFR (rereplication gene)
Bartkowiak, K. et al.	2015	Disseminated Tumor Cells Persist in the Bone Marrow of Breast Cancer Patients through Sustained Activation of the Unfolded Protein Response	*Cancer Res*	1		BC-M1, LC-M1, PC-E1, MCF-7, Hs578t, MDA-MB-231, MDA-MB-468	GRP78
Han, K. S. et al.	2015	Inhibition of endoplasmic reticulum chaperone protein glucose-regulated protein 78 potentiates anti-angiogenic therapy in renal cell carcinoma through inactivation of the PERK/eIF2alpha pathway	*Oncotarget*	1		Caki-1, Caki-2	GRP78, eIF2a
Hotokezaka, Y. et al.	2015	GSK-3beta-dependent downregulation of gamma-taxilin and alphaNAC merge to regulate ER stress responses	*Cell Death Dis*	1		HeLa	GSK-3B, γ-taxilin, αNAC
Koizume, S. et al.	2015	Lipid starvation and hypoxia synergistically activate ICAM1 and multiple genes in an Sp1-dependent manner to promote the growth of ovarian cancer	*Mol Cancer*	1		Human ovarian cancer tissue samples	ICAM1, Sp1, HIF1a, mTOR, TNFa-NFkB
Nagelkerke, A. et al.	2015	Hypoxic regulation of the PERK/ATF4/LAMP3-arm of the unfolded protein response in head and neck squamous cell carcinoma	*Head Neck*	<0.1		UT-SCC	PERK/eIF2α/ATF4/LAMP3
Notte, A. et al.	2015	Taxol-induced unfolded protein response activation in breast cancer cells exposed to hypoxia: ATF4 activation regulates autophagy and inhibits apoptosis	*Int J Biochem Cell Biol*	1		MDA-MB-231, T47D	ATF4, PERK, ATF6, IRE1
Shen, X. et al.	2015	The unfolded protein response potentiates epithelial-to-mesenchymal transition (EMT) of gastric cancer cells under severe hypoxic conditions	*Med Oncol*	0.1	1	HGC27, MGC803	PERK, ATF4, ATF6, TGFβ, Akt
Vandewynckel, Y. et al.	2015	Modulation of the unfolded protein response impedes tumor cell adaptation to proteotoxic stress: a PERK for hepatocellular carcinoma therapy	*Hepatol Int*	1		HepG2, BWTG3, Hepa1-6	PERK, IRE1, CHOP, ATF6
Verras, M. et al.	2015	WNT16-expressing Acute Lymphoblastic Leukemia Cells are Sensitive to Autophagy Inhibitors after ER Stress Induction	*Anticancer Res*	<0.01		RKO	WNT-16
Zheng, X. et al.	2015	Attenuation of oxygen fluctuation-induced endoplasmic reticulum stress in human lens epithelial cells	*Exp Ther Med*	0	1	hLECs	Nrf2, Keap1

Zhang, N. et al.	2015	Hypoxia-induced autophagy promotes human prostate stromal cells survival and ER-stress	*Biochem Biophys Res Commun*	1.0		WPMY-1	CHOP
Bi, X. et al.	2015	Acetylcholine ameliorates endoplasmic reticulum stress in endothelial cells after hypoxia/reoxygenation via M3 AChR-AMPK signaling	*Cell Cycle*	1		HUVEC	Acetycholine
Jeong, K. et al.	2015	Hypoxia induces cyclophilin B through the activation of transcription factor 6 in gastric adenocarcinoma cells	*Oncol Lett*	0.1		AGS	CypB, ATF6
Kukita, K. et al.	2015	Cancer-Associated Oxidase ERO1-alpha Regulates the Expression of MHC Class I Molecule via Oxidative Folding	*J Immunol*	1.0		SW480, MCF-7, MIAPaCa2, HCT15, HCT116, HT29	ERO1α, PDI
Burrows, N. et al.	2016	Phosphatidylinositide 3-kinase (PI3K) and PI3K-related kinase (PIKK) activity contributes to radioresistance in thyroid carcinomas	*Oncotarget*	0		FTC-133, 8505c	PI3K, PIKK, ATM, ATR
Di, K. et al.	2016	Mitochondrial Lon is over-expressed in high-grade gliomas, and mediates hypoxic adaptation: potential role of Lon as a therapeutic target in glioma	*Oncotarget*	1		U-251	Mitochondrial Lon
Dukel, M. et al.	2016	The Breast Cancer Tumor Suppressor TRIM29 Is Expressed via ATM-dependent Signaling in Response to Hypoxia	*J Biol Chem*	1		SKBr3, MDA-MB-468	TRIM29, ATM
Feng, H. et al.	2016	Hypoxia-induced autophagy as an additional mechanism in human osteosarcoma radioresistance	*J Bone Oncol*	1		Human osteosarcoma tissue samples	LC3, HIF, ROS
Garcia-Limones, C. et al.	2016	CHK2 stability is regulated by the E3 ubiquitin ligase SIAH2	*Oncogene*	1		HEK-293T, HeLa, U2OS, A549	CHK2, SIAH2
Hasvold, G. et al.	2016	Hypoxia-induced alterations of G2 checkpoint regulators	*Mol Oncol*	0.2		U2OS	CDK
Janaszak-Jasiecka, A. et al.	2016	miR-429 regulates the transition between Hypoxia-Inducible Factor (HIF)1A and HIF3A expression in human endothelial cells	*Sci Rep*	1		HUVEC	HIF1A, HIF3A, mir-429, DDIT4
Jiang, Y. et al.	2016	Hypoxia Potentiates the Radiation-Sensitizing Effect of Olaparib in Human Non-Small Cell Lung Cancer Xenografts by Contextual Synthetic Lethality	*Int J Radiat Oncol Biol Phys*	1		NSCLC Calu-6, Calu-3	PARP, yH2AX, RAD51
Liu, S. et al.	2016	Intermittent hypoxia reduces microglia proliferation and induces DNA damage in vitro	*Iran J Basic Med Sci*	1		BV2	Cyclin D1, cyclin E2, p53
Ma, D. et al.	2016	GADD45beta induction by S-adenosylmethionine inhibits hepatocellular carcinoma cell proliferation during acute ischemia-hypoxia	*Oncotarget*	1		HepG2, Hep3B	GADD45β
Memon, D. et al.	2016	Hypoxia-driven splicing into noncoding isoforms regulates the DNA damage response	*NPJ Genom Med*	1		HCT116	HDAC6, TP53BP1
Olcina, M. et al.	2016	H3K9me3 facilitates hypoxia-induced p53-dependent apoptosis through repression of APAK	*Oncogene*	0.1	1	RKO	SETDB1, APAK, P53
Wen, L. et al.	2016	Regulation of Multi-drug Resistance in hepatocellular carcinoma cells is TRPC6/Calcium Dependent	*Sci Rep*	1		Huh7, HepG2	TRPC6, STAT3
Yu, Z. et al.	2016	Tumor-Derived Factors and Reduced p53 Promote Endothelial Cell Centrosome Over-Duplication	*PLoS One*	2		HUVEC	p53, BMP proteins, SMAD, IL8
Zhou, F. et al.	2016	Regulation of hypoxia-inducible factor-1alpha, regulated in development and DNA damage response-1 and	*Placenta*	1		BeWo	HIF-1α-REDD1-mTOR

		mammalian target of rapamycin in human placental BeWo cells under hypoxia				
Chang, E. et al.	2016	Heterogenous ribonucleoprotein A18 (hnRNP A18) promotes tumor growth by increasing protein translation of selected transcripts in cancer cells	*Oncotarget*	0.5	HEMa-LP, MDA-MB-231	A18, ATR, RPA, TRX, HIF-1α
Leszczynska, K. et al.	2016	Mechanisms and consequences of ATMIN repression in hypoxic conditions: roles for p53 and HIF-1	*Sci Rep*	0	RKO	p53, HIF1α, ATMIN
Alnasser, H. et al.	2016	Requirement of clusterin expression for prosurvival autophagy in hypoxic kidney tubular epithelial cells	*Am J Physiol Renal Physiol*	1	HKC-8	Clusterin (Clu)
Bonnet-Magnaval, F. et al.	2016	Hypoxia and ER stress promote Staufen1 expression through an alternative translation mechanism	*Biochem Biophys Res Commun*	1	HeLa, HEK293	Staufen1, PERK
Burrows, N. et al.	2016	Hypoxia-induced nitric oxide production and tumour perfusion is inhibited by pegylated arginine deiminase (ADI-PEG20)	*Sci Rep*	1	HCT116, UMUC3	ADI-PEG20, HIF1, HIF2, VEGF, mTORC1, ASS1
Grandi, A. et al.	2016	ERMP1, a novel potential oncogene involved in UPR and oxidative stress defense, is highly expressed in human cancer	*Oncotarget*	1	SK-BR-3, MCF7	ERMP1, Nrf2, HIF1α
Kikuchi, D. et al.	2016	CREB is activated by ER stress and modulates the unfolded protein response by regulating the expression of IRE1alpha and PERK	*Biochem Biophys Res Commun*	1	MDA-MB231, MCF7	CREB, IRE1α, PERK
Liu, Y. et al.	2016	XBP1 silencing decreases glioma cell viability and glycolysis possibly by inhibiting HK2 expression	*J Neurooncol*	1	U87, U251	XBP1, HK2
Vandewynckel, Y. et al.	2016	Placental growth factor inhibition modulates the interplay between hypoxia and unfolded protein response in hepatocellular carcinoma	*BMC Cancer*	1	HepG2, Huh7	PlGF, PERK, IRE1
Xu, M. et al.	2016	Inhibition of the mitochondrial unfolded protein response by acetylcholine alleviated hypoxia/reoxygenation-induced apoptosis of endothelial cells	*Cell Cycle*	1	HUVECs	Acetylcholine, M3AChR
Yoo, J. et al..	2016	Differential sensitivity of hepatocellular carcinoma cells to suppression of hepatocystin transcription under hypoxic conditions	*J Bioenerg Biomembr*	1	Huh-7, SNU-761, and SNU-3058	PRKCSH
Xie, Y. et al.	2016	Protective effect of mild endoplasmic reticulum stress on radiation-induced bystander effects in hepatocyte cells	*Sci Rep*	1.0	HepG2, HL-7702	BiP-PERK-p-eIF2α
Lai, M. et al.	2016	Hypoxia Induces Autophagy through Translational Up-Regulation of Lysosomal Proteins in Human Colon Cancer Cells	*PLoS One*	1.0	HCT116	PERK, mTOR, LC3, P62
Lakhter, A. et al.	2016	Golgi Associated HIF1a Serves as a Reserve in Melanoma Cells	*J Cell Biochem*	1.0	MEL526, PC3, HT108, RPMI 8322, MEL2664	Golgi-associated HIF1α
Chen, P. et al.	2017	Amphetamines promote mitochondrial dysfunction and DNA damage in pulmonary hypertension	*JCI Insight*	0.5	Human PAECs	Sirtuin-1, HIF
Costa, V. et al.	2017	MiR-675-5p supports hypoxia induced epithelial to mesenchymal transition in colon cancer cells	*Oncotarget*	1	SW620	miR-675-5p, DDB2, HIF
Foskolou, I. et al.	2017	Ribonucleotide Reductase Requires Subunit Switching in Hypoxia to Maintain DNA Replication	*Mol Cell*	0.1 2	RKO	RRM1/RRM2B
Han, J. et al.	2017	Hypoxia is a Key Driver of Alternative Splicing in Human Breast Cancer Cells	*Sci Rep*	1	MCF7, MDA-MB-231, HeLa, SKN-BE-2	RNAseq data

Hauth, F. et al.	2017	Cell-line dependent effects of hypoxia prior to irradiation in squamous cell carcinoma lines	*Clin Transl Radiat Oncol*	0.1	1	SKX, FaDu, UT SCC-5,	RAD51, DNA PK, ATM
Jongen, J. et al.	2017	Downregulation of DNA repair proteins and increased DNA damage in hypoxic colon cancer cells is a therapeutically exploitable vulnerability	*Oncotarget*	0.1		L145, L169	RAD51, KU70 and RIF1, GPx2
Lutzkendorf, J. et al.	2017	Resistance for Genotoxic Damage in Mesenchymal Stromal Cells Is Increased by Hypoxia but Not Generally Dependent on p53-Regulated Cell Cycle Arrest	*PLoS One*	0.5		hMSCs	p53, p21
Saki, M. et al.	2017	EGFR Mutations Compromise Hypoxia-Associated Radiation Resistance through Impaired Replication Fork-Associated DNA Damage Repair	*Mol Cancer Res*	0.1		A549, H820, HCC827, H1975	EGFR, RAD50
Wu, M. et al.	2017	miR-25/93 mediates hypoxia-induced immunosuppression by repressing cGAS	*Nat Cell Biol*	1		MCF7	miR25/93, cGAS, NCOA3
Zhang, H. et al.	2017	Combination of betulinic acid and chidamide inhibits acute myeloid leukemia by suppression of the HIF1alpha pathway and generation of reactive oxygen species	*Oncotarget*	1.0		Kasumi-1, HL-60, THP-1	HIF, SOD2
Zhao, H. et al.	2017	2-Methoxyestradiol enhances radiosensitivity in radioresistant melanoma MDA-MB-435R cells by regulating glycolysis via HIF-1alpha/PDK1 axis	*Int J Oncol*	1		MDA-MB-435S	SOD2, HIF, VEGF, ROS
Freis, P. et al.	2017	mTOR inhibitors activate PERK signaling and favor viability of gastrointestinal neuroendocrine cell lines	*Oncotarget*	1		MCF-7	mTOR, PERK
Zhou, Y. et al.	2017	Downregulation of microRNA199a5p protects cardiomyocytes in cyanotic congenital heart disease by attenuating endoplasmic reticulum stress	*Mol Med Rep*	1		Human cardiac myocytes	microRNA-199a-5p, ATF6, GRP78
Yi, H. et al.	2017	PERK/eIF2alpha contributes to changes of insulin signaling in HepG2 cell induced by intermittent hypoxia	*Life Sci*	1		HepG2	PERK, eIF2α, insulin signalling, AKT
Bischoff, F. et al.	2017	Identification and Functional Characterization of Hypoxia-Induced Endoplasmic Reticulum Stress Regulating lncRNA (HypERlnc) in Pericytes	*Circ Res*	1.0		Human cardiac tissue samples	HypERlnc
Cuomo, F. et al.	2018	Pro-inflammatory cytokines activate hypoxia-inducible factor 3alpha via epigenetic changes in mesenchymal stromal/stem cells	*Sci Rep*	1		hMSCs	HIF3A, NF-κB
Eales, K. et al.	2018	Verteporfin selectively kills hypoxic glioma cells through iron-binding and increased production of reactive oxygen species	*Sci Rep*	1		U87, U343	YAP
Nakamura, H. et al.	2018	Differentiated embryo chondrocyte plays a crucial role in DNA damage response via transcriptional regulation under hypoxic conditions	*PLoS One*	1		HSC-2, HepG2	DEC1, DEC2, HIF
Yin, H. et al.	2018	The involvement of regulated in development and DNA damage response 1 (REDD1) in the pathogenesis of intervertebral disc degeneration	*Exp Cell Res*	1		Human NP cells	REDD1, NF-κB
Anusornvongchai, T. et al.	2018	Palmitate deranges erythropoietin production via transcription factor ATF4 activation of unfolded protein response	*Kidney Int*	1		HepG2	ATF4, EPO
Ivanova, I. et al.	2018	PERK/eIF2alpha signaling inhibits HIF-induced gene expression during the unfolded protein response via YB1-dependent regulation of HIF1alpha translation	*Nucleic Acids Res*	1		PC-3, U2OS, MCF7, COV-434	PERK/eIF2α, HIF1, YB-1

Xu, G. et al.	2018	Autosomal dominant retinitis pigmentosa-associated gene PRPF8 is essential for hypoxia-induced mitophagy through regulating ULK1 mRNA splicing	*Autophagy*	1		HeLa	PRPF8, mitophagy, ULK1
Tang, C. et al.	2018	Montelukast inhibits hypoxia inducible factor-1alpha translation in prostate cancer cells	*Cancer Biol Ther*	1.0		PC3, PC3-HRE-LUC, LNCaP	HIF, PERK, eIF2α
Timalsina, S. et al.	2018	Chemical compounds that suppress hypoxia-induced stress granule formation enhance cancer drug sensitivity of human cervical cancer HeLa cells	*J Biochem*	0.5		HeLa	PERK, eIF2α, stress granules
Zhang, J. et al.	2018	Secretory kinase Fam20C tunes endoplasmic reticulum redox state via phosphorylation of Ero1alpha	*EMBO J*	0.1		HeLa	FAM20C, ERO1α
Bousquet, P. et al.	2019	Markers of Mitochondrial Metabolism in Tumor Hypoxia, Systemic Inflammation, and Adverse Outcome of Rectal Cancer	*Transl Oncol*	0.2		HCT-116, HT-29, LoVo	Inflammatory markers
Cowman, S. et al.	2019	Decrease of Nibrin expression in chronic hypoxia is associated with hypoxia-induced chemoresistance in some brain tumour cells	*BMC Cancer*	1		U87	MRE11/RADAD50/NBS, p53
Foltyn, M. et al.	2019	The physiological mTOR complex 1 inhibitor DDIT4 mediates therapy resistance in glioblastoma	*Br J Cancer*	0.1		LNT-229, LN-308, G55	mmTOR, DDIT4, TSC1/2
Giovannini, S. et al.	2019	Synthetic lethality between BRCA1 deficiency and poly(ADP-ribose) polymerase inhibition is modulated by processing of endogenous oxidative DNA damage	*Nucleic Acids Res*	1		A2780	BRCA1, PARP, OGG1
Goto, T. et al.	2019	WEE1 inhibition enhances sensitivity to hypoxia/reoxygenation in HeLa cells	*J Radiat Res*	0.1		HeLa	WEE1, CHK1, CHK2, BRCA1
Gottgens, E. et al.	2019	Inhibition of CDK4/CDK6 Enhances Radiosensitivity of HPV Negative Head and Neck Squamous Cell Carcinomas	*Int J Radiat Oncol Biol Phys*	0.1	1	UT-SCC	CDK4, CDK6, p16
Krokidis, M. et al.	2019	Purine DNA Lesions at Different Oxygen Concentration in DNA Repair-Impaired Human Cells (EUE-siXPA)	*Cells*	1		EUE-siXPA, EUE-pBD650	XPA
Li, M. et al.	2019	Distinct APE1 Activities Affect the Regulation of VEGF Transcription Under Hypoxic Conditions	*Comput Struct Biotechnol J*	1		HUVEC	APE1, VEGF
Mongiardi, M. et al.	2019	Gene expression profiling of hypoxic response in different models of senescent endothelial cells	*Aging Clin Exp Res*	2		HUVEC	RNA seq targets
Okuyama, K. et al.	2019	Mieap-induced accumulation of lysosomes within mitochondria (MALM) regulates gastric cancer cell invasion under hypoxia by suppressing reactive oxygen species accumulation	*Sci Rep*	1		58As9	Mieap, BNIP3 and BNIP3L, cathepsin D
Pan, W. et al.	2019	Hypoxia-induced microRNA-191 contributes to hepatic ischemia/reperfusion injury through the ZONAB/Cyclin D1 axis	*Cell Death Differ*	1		LO2	microRNA-191, ZONAB/Cyclin D1
Peng, M. et al.	2019	Intracellular citrate accumulation by oxidized ATM-mediated metabolism reprogramming via PFKP and CS enhances hypoxic breast cancer cell invasion and metastasis	*Cell Death Dis*	1		BT549, Hs578T	ATM, PFKP, AKT/ERK/MMP2/9
Schmit, K. et al.	2019	Characterization of the role of TMEM45A in cancer cell sensitivity to cisplatin	*Cell Death Dis*	1		SQD9, Cal 27	TMEM45A
Seong, M. et al.	2019	Hypoxia-induced regulation of mTOR signaling by miR-7 targeting REDD1	*J Cell Biochem*	1		HeLa	REDD1, mTOR, mir-7

Sun, K. et al.	2019	Oxidized ATM-mediated glycolysis enhancement in breast cancer-associated fibroblasts contributes to tumor invasion through lactate as metabolic coupling	*EBioMedicine*	1	CAFS	GLUT1, PKM2, PI3K/AKT, ATM
Van den Bossche, J. et al.	2019	In vitro study of the Polo-like kinase 1 inhibitor volasertib in non-small-cell lung cancer reveals a role for the tumor suppressor p53	*Mol Oncol*	0	A549, NCI-H1975	p53, Plk1
Zhihua, Y. et al.	2019	Hypoxia decreases macrophage glycolysis and M1 percentage by targeting microRNA-30c and mTOR in human gastric cancer	*Cancer Sci*	1	THP-1	miR30-c, mTOR
Cao, X. et al.	2019	The IRE1alpha-XBP1 pathway function in hypoxia-induced pulmonary vascular remodeling, is upregulated by quercetin, inhibits apoptosis and partially reverses the effect of quercetin in PASMCs	*Am J Transl Res*	1	Human PASMCs	Quercetin, IRE1/XBP1,
Jaud, M. et al.	2019	The PERK Branch of the Unfolded Protein Response Promotes DLL4 Expression by Activating an Alternative Translation Mechanism	*Cancers (Basel)*	1	HeLa, NIH3T3, eIF2αS51A MEF	PERK, DLL4
Liew, L. et al.	2019	Hypoxia-Activated Prodrugs of PERK Inhibitors	*Chem Asian J*	0	HCT116	PERK
Xia, Z. et al.	2019	Hypoxic ER stress suppresses beta-catenin expression and promotes cooperation between the transcription factors XBP1 and HIF1alpha for cell survival	*J Biol Chem*	1%	RKO	β-catenin, HIF1α, XBP1
Xin, L. et al.	2019	4-phenylbutyric acid attenuates endoplasmic reticulum stress-mediated apoptosis and protects the hepatocytes from intermittent hypoxia-induced injury	*Sleep Breath*	1.5	L02	PERK-eIF2α-ATF4-CHOP
Bommi, P. et al.	2020	NER-factor DDB2 regulates HIF1alpha and hypoxia-response genes in HNSCC	*Oncogene*	1	SCC9, SCC15	DDB2, HIF
Curro, M. et al.	2020	Hypoxia-Dependent Expression of TG2 Isoforms in Neuroblastoma Cells as Consequence of Different MYCN Amplification Status	*Int J Mol Sci*	1	SH-SY5Y	TG2, MYCN
Fu, Z. et al.	2020	HIF-1alpha-BNIP3-mediated mitophagy in tubular cells protects against renal ischemia/reperfusion injury	*Redox Biol*	1	HK2, CRL-2190	TOMM20, COX IV, BNIP3
Garcia-Venzor, A. et al.	2020	LncMat2B regulated by severe hypoxia induces cisplatin resistance by increasing DNA damage repair and tumor-initiating population in breast cancer cells	*Carcinogenesis*	0.1	MCF-7, MDA-MB-231	lncMat2B
Guo, Q. et al.	2020	ATM-CHK2-Beclin 1 axis promotes autophagy to maintain ROS homeostasis under oxidative stress	*EMBO J*	1.0	H1299, HEK293T, HEK293, HeLa	ATM-CHK2-Beclin 1
Hassan Venkatesh, G. et al.	2020	Hypoxia increases mutational load of breast cancer cells through frameshift mutations	*Oncoimmunology*	1	MCF-7, MDA-MB-231	Gene mutations
Huan, L. et al.	2020	Hypoxia induced LUCAT1/PTBP1 axis modulates cancer cell viability and chemotherapy response	*Mol Cancer*	1	HEK-293 T, HCT-116, RKO, LoVo	LUCAT1/PTBP1
Liu, Y. et al.	2020	Oroxylin A reverses hypoxia-induced cisplatin resistance through inhibiting HIF-1alpha mediated XPC transcription	*Oncogene*	1	H460, A549, 95D, PC9, HCC827, H1975	XPC, HIF
Luo, A. et al.	2020	Proteome dynamics analysis identifies functional roles of SDE2 and hypoxia in DNA damage response in prostate cancer cells	*NAR Cancer*	1	DU145, PC3	SDE2, CDT2, HIF, PCNA

Ma, Y. et al.	2020	Loss of heterozygosity for Kras(G12D) promotes REDD1-dependent, non-canonical glutamine metabolism in pancreatic ductal adenocarcinoma	*Biochem Biophys Res Commun*	1	PDACs	KRAS, REDD1
Spina, R. et al.	2020	MCT4 regulates de novo pyrimidine biosynthesis in GBM in a lactate-independent manner	*Neurooncol Adv*	1	HSR-GBM1, HSR040821	MCT4
Torrisi, F. et al.	2020	SRC Tyrosine Kinase Inhibitor and X-rays Combined Effect on Glioblastoma Cell Lines	*Fundam Clin Pharmacol*	1	U251-MG, U87-MG	c-SRC
Xia, X.;Wang, Q.;Ye, T.;Liu, Y.;Liu, D.;Song, S.;Zheng, C.	2020	NRF2/ABCB1-mediated efflux and PARP1-mediated dampening of DNA damage contribute to doxorubicin resistance in chronic hypoxic HepG2 cells	*Fundam Clin Pharmacol*	1	HepG2	NRF2/ABCB1
Yao, T. et al.	2020	ALDH-1-positive cells exhibited a radioresistant phenotype that was enhanced with hypoxia in cervical cancer	*BMC Cancer*	1	HeLa, Siha	ALDH, CHK1
Zhao, X. et al.	2020	Long noncoding RNA NORAD regulates angiogenesis of human umbilical vein endothelial cells via miR5903p under hypoxic conditions	*Mol Med Rep*	1	HUVEC	lncRNA NORAD/miR-590-3p
Diaz, M. et al.	2020	Caveolin-1 suppresses tumor formation through the inhibition of the unfolded protein response	*Cell Death Dis*	1	MDA-MB-231	Caveolin-1
Lee, Y. et al.	2020	Androgen-induced expression of DRP1 regulates mitochondrial metabolic reprogramming in prostate cancer	*Cancer Lett*	0.5	LNCaP	DRP1
Moszynska, A. et al.	2020	IRE1 Endoribonuclease Activity Modulates Hypoxic HIF-1alpha Signaling in Human Endothelial Cells	*Biomolecules*	0.9	HUVEC	IRE1, HIF1α
Zhang, J. et al.	2020	Endoplasmic Reticulum stress-dependent expression of ERO1L promotes aerobic glycolysis in Pancreatic Cancer	*Theranostics*	1	AsPC-1, BxPC-3, Capan-2, CFP AC-1, MiaPaCa-2, P ANC-1, Patu8988	ERO1L
Zheng, H. et al.	2020	Hypoxia Induces Growth Differentiation Factor 15 to Promote the Metastasis of Colorectal Cancer via PERK-eIF2alpha Signaling	*Biomed Res Int*	1.0	HT29, SW480	GDF15, PERK/eIF2α
Soni, H. et al.	2020	PERK-mediated expression of peptidylglycine alpha-amidating monooxygenase supports angiogenesis in glioblastoma	*Oncogenesis*	1	LN308, LN229	PAM, PERK, HIF

Table S3. **Genes identified as potential links between the UPR and DDR in hypoxia**

From the systematic review, a list of genes was identified as playing key roles in the hypoxia-induced UPR (coloured in green) or the hypoxia-induced DDR (blue). Each gene has previously been linked to the other stress pathway in normoxia, for example LAMP3 was highlighted in the systematic review as being involved in the hypoxic UPR, but it has also been linked to the DDR in normoxia. Conversely, JMJD2B was highlighted in the systematic review has being involved in the hypoxic DDR but has only been linked to the UPR in normoxia.

**Gene**	**Regulation by the UPR**	**Oxygen concentration investigated (% O_2)**	**References**
LAMP3	PERK/eIF2α/ATF4	0.1, 1.0	(1–3)
ULK1	PERK/eIF2α/ATF4	<0.02	(4)
TRIB3	PERK/eIF2α/ATF4	0.1 0.2, 0.5	(5)
CHOP	PERK/eIF2α/ATF4	1.0	(1,6–14)
NOXA	PERK/eIF2α/ATF4	1.0	(15)
NORAD	PERK/eIF2α/ATF4	1.0	(16)
SIAH1/2	PERK/eIF2α/ATF4 and IRE1α/XBP1	1.0	(17–20)
DYRK2	PERK/eIF2α/ATF4	1.0	(18)
HIPK2	PERK/eIF2α/ATF4	0.2, 1.0, 2.0	(17,21)
CREB	PERK/eIF2α/ATF4	1.0	(22)
NUPR1	PERK/eIF2α/ATF4	0.2	(23)
NRF2	PERK/eIF2α/ATF4	0, 1.0	(24–28)
GSK-3B	PERK/eIF2α/ATF4	1.0	(29)
STAU1	PERK/eIF2α/ATF4 and interacts with XBP1	1.0	(30)
GADD45B	PERK/eIF2α/ATF4	1.0	(31)
C-SRC	IRE1α/XBP1	1.0	(32)
HK2	IRE1α/XBP1	1.0	(33)
CAV1	IRE1α/XBP1	1.0	(34)
CYPB	ATF6	0.1	(14,35)
CLU	GRP78 binding	1.0	(36)
IGFBP-3	GRP78 binding	1.0	(37)
SP1	General ER stress induced	1.0	(38)
**Gene**	**Regulation by the DDR**	**Oxygen concentration investigated (% O_2)**	**References**
JMJD2B	Activates hypoxic DDR via ATM-RAD3 pathway	1.0	(39)

References

1. Mujcic H, Nagelkerke A, Rouschop KMA, Chung S, Chaudary N, Span PN, et al. Hypoxic activation of the PERK/eIF2α arm of the unfolded protein response promotes metastasis through induction of LAMP3. Clin Cancer Res. 2013 Nov 15;19(22):6126–37.

2. Nagelkerke A, Sweep FCGJ, Stegeman H, Grénman R, Kaanders JHAM, Bussink J, et al. Hypoxic regulation of the PERK/ATF4/LAMP3-arm of the unfolded protein response in head and neck squamous cell carcinoma. Head Neck. 2015 Jun;37(6):896–905.

3. Nagelkerke A, Bussink J, Mujcic H, Wouters BG, Lehmann S, Sweep FCGJ, et al. Hypoxia stimulates migration of breast cancer cells via the PERK/ATF4/LAMP3-arm of the unfolded protein response. Breast Cancer Res. 2013 Jan 7;15(1):R2.

4. Schaaf MBE, Cojocari D, Keulers TG, Jutten B, Starmans MH, de Jong MC, et al. The autophagy associated gene, ULK1, promotes tolerance to chronic and acute hypoxia. Radiother Oncol. 2013 Sep;108(3):529–34.

5. Wennemers M, Bussink J, Scheijen B, Nagtegaal ID, van Laarhoven HW, Raleigh JA, et al. Tribbles homolog 3 denotes a poor prognosis in breast cancer and is involved in hypoxia response. Breast Cancer Res. 2011 Aug;13(4):R82.

6. Rouschop KM, Dubois LJ, Keulers TG, van den Beucken T, Lambin P, Bussink J, et al. PERK/eIF2α signaling protects therapy resistant hypoxic cells through induction of glutathione synthesis and protection against ROS. Proc Natl Acad Sci U S A. 2013 Mar 19;110(12):4622–7.

7. Pi L, Li X, Song Q, Shen Y, Lu X, Di B. Knockdown of glucose-regulated protein 78 abrogates chemoresistance of hypopharyngeal carcinoma cells to cisplatin induced by unfolded protein in response to severe hypoxia. Oncol Lett. 2014 Mar;7(3):685–92.

8. Vandewynckel Y-P, Laukens D, Bogaerts E, Paridaens A, Van den Bussche A, Verhelst X, et al. Modulation of the unfolded protein response impedes tumor cell adaptation to proteotoxic stress: a PERK for hepatocellular carcinoma therapy. Hepatol Int. 2015 Jan;9(1):93–104.

9. Xu M, Bi X, He X, Yu X, Zhao M, Zang W. Inhibition of the mitochondrial unfolded protein response by acetylcholine alleviated hypoxia/reoxygenation-induced apoptosis of endothelial cells. Cell Cycle. 2016 May 18;15(10):1331–43.

10. Yoo J-J, Lee DH, Cho Y, Cho EJ, Lee J-H, Yu SJ, et al. Differential sensitivity of hepatocellular carcinoma cells to suppression of hepatocystin transcription under hypoxic conditions. J Bioenerg Biomembr. 2016 Dec;48(6):581–90.

11. Petry A, Zhang Z, Trautz B, Rieß F, Görlach A. Cross Talk Between p22phox and ATF4 in the Endothelial Unfolded Protein Response. Antioxid Redox Signal. 2019 Jan 1;30(1):40–55.

12. Zheng H, Wu Y, Guo T, Liu F, Xu Y, Cai S. Hypoxia Induces Growth Differentiation Factor 15 to Promote the Metastasis of Colorectal Cancer via PERK-eIF2 *α* Signaling. BioMed Research International. 2020 Jan 29;2020:1–12.

13. Zhang N, Ji N, Jiang W-M, Li Z-Y, Wang M, Wen J-M, et al. Hypoxia-induced autophagy promotes human prostate stromal cells survival and ER-stress. Biochemical and Biophysical Research Communications. 2015 Sep 4;464(4):1107–12.

14. Jeong K, Kim H, Kim K, Kim S-J, Hahn B-S, Jahng G-H, et al. Cyclophilin B is involved in p300-mediated degradation of CHOP in tumor cell adaptation to hypoxia. Cell Death Differ. 2014 Mar;21(3):438–50.

15. Sermeus A, Genin M, Maincent A, Fransolet M, Notte A, Leclere L, et al. Hypoxia-Induced Modulation of Apoptosis and BCL-2 Family Proteins in Different Cancer Cell Types. PLoS One [Internet]. 2012 Nov 5 [cited 2021 Feb 21];7(11).

16. Zhao X, Wei X, Wang X, Qi G. Long non-coding RNA NORAD regulates angiogenesis of human umbilical vein endothelial cells via miR-590-3p under hypoxic conditions. Mol Med Rep. 2020 Jun;21(6):2560–70.

17. Moehlenbrink J, Bitomsky N, Hofmann TG. Hypoxia suppresses chemotherapeutic drug-induced p53 Serine 46 phosphorylation by triggering HIPK2 degradation. Cancer Lett. 2010 Jun 1;292(1):119–24.

18. Pérez M, García-Limones C, Zapico I, Marina A, Schmitz ML, Muñoz E, et al. Mutual regulation between SIAH2 and DYRK2 controls hypoxic and genotoxic signaling pathways. J Mol Cell Biol. 2012 Oct;4(5):316–30.

19. Adam MG, Matt S, Christian S, Hess-Stumpp H, Haegebarth A, Hofmann TG, et al. SIAH ubiquitin ligases regulate breast cancer cell migration and invasion independent of the oxygen status. Cell Cycle. 2015;14(23):3734–47.

20. García-Limones C, Lara-Chica M, Jiménez-Jiménez C, Pérez M, Moreno P, Muñoz E, et al. CHK2 stability is regulated by the E3 ubiquitin ligase SIAH2. Oncogene. 2016 Aug 18;35(33):4289–301.

21. Tong Y, Li Q-G, Xing T-Y, Zhang M, Zhang J-J, Xia Q. HIF1 regulates WSB-1 expression to promote hypoxia-induced chemoresistance in hepatocellular carcinoma cells. FEBS Lett. 2013 Aug 19;587(16):2530–5.

22. Kikuchi D, Tanimoto K, Nakayama K. CREB is activated by ER stress and modulates the unfolded protein response by regulating the expression of IRE1α and PERK. Biochemical and Biophysical Research Communications. 2016 Jan 8;469(2):243–50.

23. Hamidi T, Cano CE, Grasso D, Garcia MN, Sandi MJ, Calvo EL, et al. Nupr1-Aurora Kinase A Pathway Provides Protection against Metabolic Stress-Mediated Autophagic-Associated Cell Death. Clin Cancer Res. 2012 Oct 1;18(19):5234–46.

24. Xia X, Wang Q, Ye T, Liu Y, Liu D, Song S, et al. NRF2/ABCB1-mediated efflux and PARP1-mediated dampening of DNA damage contribute to doxorubicin resistance in chronic hypoxic HepG2 cells. Fundam Clin Pharmacol. 2020 Feb;34(1):41–50.

25. Elanchezhian R, Palsamy P, Madson CJ, Mulhern ML, Lynch DW, Troia AM, et al. Low glucose under hypoxic conditions induces unfolded protein response and produces reactive oxygen species in lens epithelial cells. Cell Death Dis. 2012 Apr 19;3:e301.

26. Zheng X-Y, Xu J, Chen XI, Li W, Wang T-Y. Attenuation of oxygen fluctuation-induced endoplasmic reticulum stress in human lens epithelial cells. Exp Ther Med. 2015 Nov;10(5):1883–7.

27. Grandi A, Santi A, Campagnoli S, Parri M, Camilli ED, Song C, et al. ERMP1, a novel potential oncogene involved in UPR and oxidative stress defense, is highly expressed in human cancer. Oncotarget. 2016 Aug 23;7(39):63596–610.

28. Miyamoto N, Izumi H, Miyamoto R, Bin H, Kondo H, Tawara A, et al. Transcriptional regulation of activating transcription factor 4 under oxidative stress in retinal pigment epithelial ARPE-19/HPV-16 cells. Invest Ophthalmol Vis Sci. 2011 Mar 2;52(3):1226–34.

29. Hotokezaka Y, Katayama I, van Leyen K, Nakamura T. GSK-3β-dependent downregulation of γ-taxilin and αNAC merge to regulate ER stress responses. Cell Death Dis. 2015 Apr;6(4):e1719–e1719.

30. Bonnet-Magnaval F, Philippe C, Van Den Berghe L, Prats H, Touriol C, Lacazette E. Hypoxia and ER stress promote Staufen1 expression through an alternative translation mechanism. Biochem Biophys Res Commun. 2016 Oct 14;479(2):365–71.

31. Ma D, Shen B, Seewoo V, Tong H, Yang W, Cheng X, et al. GADD45β induction by S-adenosylmethionine inhibits hepatocellular carcinoma cell proliferation during acute ischemia-hypoxia. Oncotarget. 2016 May 11;7(24):37215–25.

32. Torrisi F, Minafra L, Cammarata FP, Savoca G, Calvaruso M, Vicario N, et al. SRC Tyrosine Kinase Inhibitor and X-rays Combined Effect on Glioblastoma Cell Lines. Int J Mol Sci. 2020 May 30;21(11).

33. Liu Y, Hou X, Liu M, Yang Z, Bi Y, Zou H, et al. XBP1 silencing decreases glioma cell viability and glycolysis possibly by inhibiting HK2 expression. J Neurooncol. 2016 Feb;126(3):455–62.

34. Díaz MI, Díaz P, Bennett JC, Urra H, Ortiz R, Orellana PC, et al. Caveolin-1 suppresses tumor formation through the inhibition of the unfolded protein response. Cell Death Dis. 2020 Aug 3;11(8):648.

35. Jeong K, Kim K, Kim H, Oh Y, Kim S-J, Jo Y, et al. Hypoxia induces cyclophilin B through the activation of transcription factor 6 in gastric adenocarcinoma cells. Oncol Lett. 2015 Jun;9(6):2854–8.

36. Alnasser HA, Guan Q, Zhang F, Gleave ME, Nguan CYC, Du C. Requirement of clusterin expression for prosurvival autophagy in hypoxic kidney tubular epithelial cells. American Journal of Physiology-Renal Physiology. 2015 Nov 11;310(2):F160–73.

37. Grkovic S, O'Reilly VC, Han S, Hong M, Baxter RC, Firth SM. IGFBP-3 binds GRP78, stimulates autophagy and promotes the survival of breast cancer cells exposed to adverse microenvironments. Oncogene. 2013 May;32(19):2412–20.

38. Koizume S, Ito S, Nakamura Y, Yoshihara M, Furuya M, Yamada R, et al. Lipid starvation and hypoxia synergistically activate ICAM1 and multiple genes in an Sp1-dependent manner to promote the growth of ovarian cancer. Mol Cancer. 2015 Dec;14(1):77.

39. Chen L, Fu L, Kong X, Xu J, Wang Z, Ma X, et al. Jumonji domain-containing protein 2B silencing induces DNA damage response via STAT3 pathway in colorectal cancer. British Journal of Cancer. 2014 Feb;110(4):1014–26.
